# A collaborative clinical case conference model for teaching social and behavioral science in medicine: an action research study

**DOI:** 10.1186/s12909-021-03009-8

**Published:** 2021-11-12

**Authors:** Junichiro Miyachi, Junko Iida, Yosuke Shimazono, Hiroshi Nishigori

**Affiliations:** 1 Center for Medical Education, Graduate School of Medicine, Nagoya University, 65 Tsurumai-cho Showa-ku Nagoya, Nagoya, Aichi 466-8560 Japan; 2 Azai-Higashi Clinic, Hokkaido Centre for Family Medicine, Sapporo, Hokkaido Japan; 3 Medical Education Center, Graduate School of Medicine, Kyoto University, Kyoto, Japan; 4 Faculty of Health and Welfare, Kawasaki University of Medical Welfare, Okayama, Japan; 5 Center for Global Initiatives, Osaka University, Osaka, Japan

**Keywords:** Social and behavioral sciences, Anthropology, Integration, Clinical case conference, Faculty development

## Abstract

**Background:**

Effective social and behavioral sciences teaching in medical education requires integration with clinical experience, as well as collaboration between social and behavioral sciences experts and clinical faculty. However, teaching models for achieving this integration have not been adequately established, nor has the collaboration process been described. This study aims to propose a collaborative clinical case conference model to integrate social and behavioral sciences and clinical experience. Additionally, we describe how social and behavioral science experts and clinical faculty collaborate during the development of the teaching method.

**Methods:**

A team of medical teachers and medical anthropologists planned for the development of a case conference based on action research methodology. The initial model was planned for a 3-h session, similar to a Clinicopathological Conference (CPC) structure. We evaluated each session based on field notes taken by medical anthropologists and post-session questionnaires that surveyed participants’ reactions and points of improvement. Based on the evaluation, a reflective meeting was held to discuss revisions for the next trial. We incorporated the development process into undergraduate medical curricula in clinical years and in a postgraduate and continuous professional development session for residents and certified family physicians in Japan. We repeated the plan-act-observe-reflection process more than 15 times between 2015 and 2018.

**Results:**

The development of the collaborative clinical case conference model is summarized in three phases: Quasi-CPC, Interactive, and Co-constructive with unique structures and underlying paradigms. The model successfully contributed to promoting the participants’ recognition of the clinical significance of social and behavioral sciences. The case preparation entailed unique and significant learning of how social and behavioral sciences inform clinical practice. The model development process promoted the mutual understanding between clinical faculty and anthropologists, which might function as faculty development for teachers involved in social and behavioral sciences teaching in medical education.

**Conclusions:**

The application of appropriate conference models and awareness of their underlying paradigms according to educational situations promotes the integration of social and behavioral sciences with clinical medicine education. Faculty development regarding social and behavioral sciences in medical education should focus on collaboration with scholars with different paradigmatic orientations.

**Supplementary Information:**

The online version contains supplementary material available at 10.1186/s12909-021-03009-8.

## Background

It has been widely suggested that teaching social and behavioral sciences (SBS) in medical education is necessary since it is incumbent upon clinicians to learn how to understand and address the social factors that are inextricably connected to health and disease [1, 2]. However, teaching SBS in medical education has proved difficult for a long time [3]. One difficulty pointed out in a recent systematic review is the lack of perceived clinical relevance of SBS to both medical students and clinical teachers [4]. For students, this is particularly notable when courses begin with conventional teachings, such as learning fundamental SBS theories with classical readings [5]. It is our standpoint that educators should contextualize SBS with particular relevance to clinical medicine to ensure effective attention [6]. The lack of perceived relevance of SBS for clinical teachers can be damaging to its effective teaching. First, such perceptions can lead them to discredit the value of learning SBS, which causes their students to regard it as a peripheral subject compared to biomedicine [ 7]. Second, clinical teachers may not effectively guide the integration of SBS learning objectives into a teaching method (e.g. Problem-Based Learning), which causes a tendency to downgrade SBS learning [6]. Third, clinical teachers may not demonstrate the integration of SBS into their clinical practice, which leads to a lack of SBS role modeling for medical students during their clinical rotations [ 8]. To address this lack of perceived clinical relevance, it is important to not only demonstrate that SBS entails immediate and visible clinical significance, but also to challenge and change the process by which such perceptions in relation to a particular topic (e.g., societal and structural forces) are constructed. In order to achieve this goal, it is necessary to develop an educational model that allows SBS scholars to engage in discussions with medical students and clinicians in their specific clinical experiences at all stages of pre-graduate, post-graduate, and continuous professional development (CPD). This is in line with the recommendations of current studies, which suggest that the comprehensive integration of SBS across these three stages of medical education is advisable to promote students’ effective learning and clinicians’ deeper understanding of the area [9–12].

The use of either fictional or authentic clinical cases is a common strategy for enhancing the integration of non-clinical sciences (including SBS) and clinical practice by contextualizing non-clinical science learning into clinical cases [13], or promoting reflection [14]. While previous articles have reported on curricula that apply this approach in teaching SBS [15, 16], they were mainly in the context of the preclinical years of undergraduate medical education, which deals with fictional cases (e.g., Problem-Based Learning [17]). Although there are a few studies that focused on clinical years in both the undergraduate and postgraduate settings [16, 18], they neither explored how to specifically proceed with the integration of clinical experiences with SBS, nor did they describe how the collaboration between clinical faculty and SBS experts might progress.

In the context of CPD, a recent aspirational attempt that seems to contribute to the demonstration of the clinical relevance of SBS is the “Case Studies in Social Medicine” series, wherein clinical cases are presented with a range of topics in SBS [19]. Such case studies should be attempted not only in academic journals, but also based on individual clinicians’ experiences in the CPD setting to maximize their impact. In short, medical educators need to develop opportunities for individual clinicians and medical students to learn the basics of SBS and integrate it into their clinical experience. Ensuring such opportunities will raise clinicians who can articulate SBS’s relevance through their clinical experience, which could compensate for the ineffective teaching and the lack of role modeling observed to date mentioned above. Moreover, it is expected that these clinicians will be less likely to undermine the value of SBS when they teach medical students. Thus, the development of a case conference model that facilitates the integration of clinical experiences and SBS knowledge is imperative. In summary, there is scarce research describing a teaching strategy that integrates SBS contents with authentic clinical experiences and that informs clinical practice.

The integration of SBS into clinical practice requires effective collaboration between clinical faculty and SBS scholars, which is another significant barrier for SBS experts and clinical faculty [4]. For SBS experts, it is difficult to contextualize SBS contents and theories in clinical practice. On the other hand, while clinical faculty can potentially integrate clinical practice into SBS, they do not have adequate knowledge of the latter. Accordingly, collaboration in the context of medical education has been highlighted as a necessity for clinicians, clinical teachers, and SBS experts [4]. However, previous literature has not explored how such collaboration can develop during the development of SBS teaching methods.

To address these gaps, we conducted a research project to develop a new case conference model for teaching SBS. Among the broad disciplines in SBS, this study focuses on anthropology in the context of teaching SBS in medical education. The research methodology in anthropology is expected to be potentially congruent with case-based teaching. An ethnographic approach, which is the principal research method in anthropology, involves participant observation, which usually starts with an authentic particular activity of a group of people [20]. Most anthropologists are expected to be familiar with the discussion through a description of a particular patient. The exclusiveness of ethnography and its potential familiarity with case-based teaching makes anthropology an ideal initial platform across the broad SBS disciplines in our research. We also explored the process of collaboration between clinical faculty and anthropologists because of the scarcity in the existing literature, despite it being a significant source of difficulty in integrating SBS into clinical practice.

Our research aims are as follows:

1. Establish the optimum structure of a clinical case conference model for teaching SBS contents;
2. Determine how this model is different from the conventional medical case conference;
3. Describe how SBS scholars and clinical faculty can collaborate in teaching SBS during the development of such a teaching model.

The context of this research project and the impact of the collaboration on anthropology and anthropologists have been discussed by the second and last authors elsewhere [21]. In the current article, we started by particularly focusing on the developmental process of the new case conference model and describe the differences between the conventional one and our new model. Next, we describe the dynamic process of collaboration between clinicians and anthropologists during the research project, which suggests that our model functions as faculty development as well. Finally, we elaborate on practical and theoretical implications for medical educators and medical education researchers.

## Methods

### Design

We undertook action research, which “is an iterative process in which researchers and practitioners act together in the context of an identified problem to discover and effect positive change.” [22] Unlike intervention research, which focuses on proving outcomes of interventions, action research focuses on the process of developing interventions for practical problems in the real world, reflecting in and on the process [23]. One of the goals is to extract transferable practical knowledge that transcends the local context in which the research was conducted [24, 25]. In this project, the primary research aim was to make changes to the conventional case conference model to tackle practical problems in the real world, such as difficulties in integrating SBS and clinical experiences.

An action research study consists of four iterative phases: planning, action, observation, and reflection [24, 26]. The iterative process enables researchers to describe the development process of an intervention. This feature aligned with our research aim, which was to clarify the differences between the new case conference model and the conventional one, as well as the process of collaboration between clinical faculty and SBS experts.

### Research team

The research team consisted of two clinicians (JM, a family physician and medical education researcher; and HN, a general internist and medical education researcher) and two medical anthropologists (JI and YS). Neither anthropologist had worked in the medical school prior to this project.

### Recruitment, data collection, and data analysis

In action research, unlike conventional intervention research, data collection and data analysis are part of a broader plan-observe-reflect-act cycle [24, 26]. In this section, we will explain data collection and data analysis based on the iterative cycle.

For the planning phase, we initially planned the case conference as a 3-h session with a presentation of two clinical cases by clinicians or students, followed by comments based on anthropological theories and perspectives made by the anthropologists on the cases. The structure of the case conference was almost identical to that of the Clinicopathological Conference (CPC), which consists of a clinician’s case presentation, discussion by the participants, and a definite diagnosis with some relevant commentary by pathologists. This was because the structure of the CPC was intended to integrate clinical medicine and pathology. It was expected to also be partly useful in integrating clinical medicine and the social sciences. Moreover, the structure of the CPC was a typical clinical conference that many clinicians are familiar with.

We incorporated the project into undergraduate medical curricula in clinical years, as well as in postgraduate and CPD sessions for residents and family physicians. For the undergraduate level, we introduced the case conference as a reflective component of clinical clerkship, where students reflected on and discussed their clinical experiences. In the postgraduate or CPD session, we held a workshop as part of the academic conference or seminar in which residents and certified family physicians voluntarily participated. In both settings, we recruited participants who were interested in the project using convenience sampling. Written informed consent was obtained from all participants.

We applied two methods for observing our sessions. First, the medical anthropologists from our team took field notes describing the process of the participants’ discussion. Second, we administered an open-response evaluative questionnaire in which we surveyed participants’ reactions to and perceived learning from the session and gathered their suggestions regarding the model. After each trial, the team members held a reflective meeting where they shared their observations and experiences, elaborated on lessons from the trials, and discussed points for revision for the next trial. Based on these meetings, we also discussed the features of participation and the roles of clinical faculty and anthropologists.

In the Results section, we first describe the latest version of our case conference model. Then, we describe its developmental process to clarify its difference from the conventional medical case conference. Finally, we will summarize how the collaboration progressed from the perspective of the role of clinical faculty and anthropologists.

### Ethics

Ethical approval was given by the Kyoto University Research Ethics Committee.

## Results

### Final structure of the Collaborative Clinical Case Conference (CCCC) model

From 2015 to 2018, we held 7 sessions at the undergraduate level, and 10 trials in postgraduate and CPD settings for family physicians. The cumulative number of participants were approximately 260 in undergraduate and 300 in the postgraduate and CPD settings. The number of participants per session was mostly 10–40. The number of participants, clinical teachers, and anthropologists, summary of presented cases and anthropologists’ comments in each session are shown in Supplementary material 1 and 2.

**Fig. 1:**
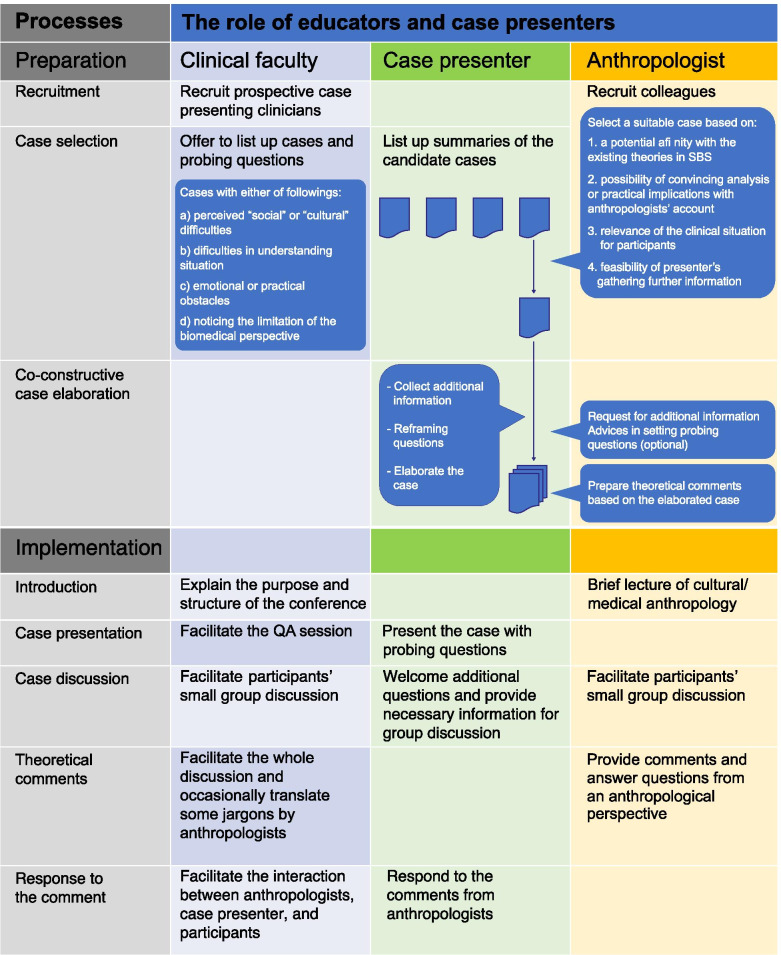
The final structure of the CCCC model

**Fig. 2:**
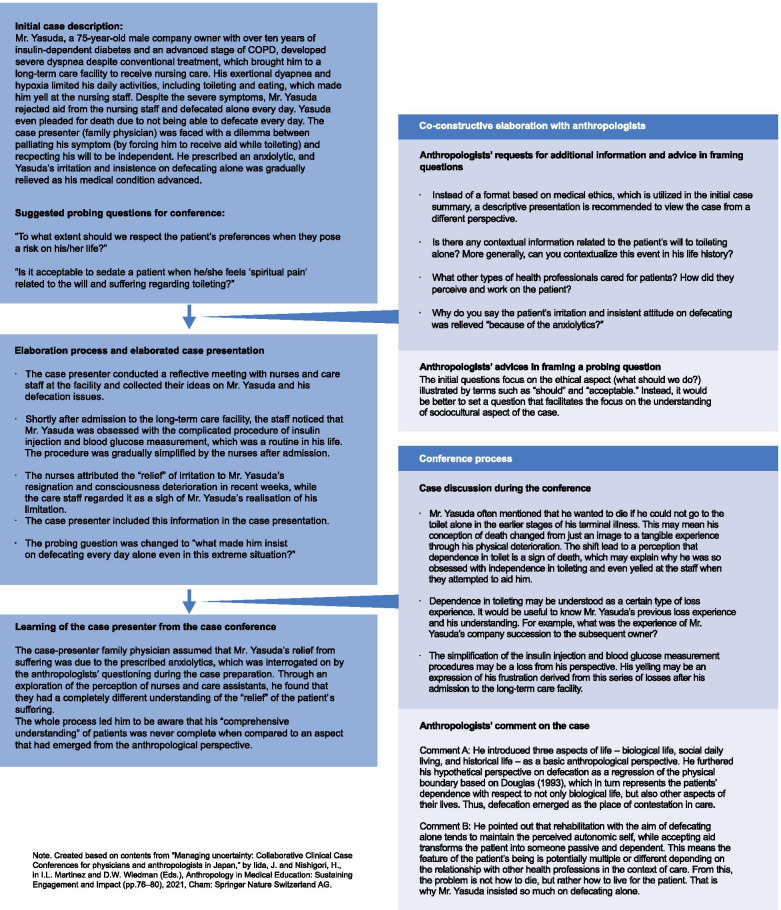
An example of the entire CCCC process

The final procedures of the conference model, what we call the collaborative clinical case conference (CCCC) model, and the role of clinical faculty, anthropologists, and case presenters are summarized in Fig. 1, which is divided into two phases: preparation and implementation. An illustrative example of how the case conference proceeded is shown in Fig. 2.

In the preparation phase of the CCCC, clinical faculty first recruited a prospective presenter, and anthropologists recruited anthropologists who wished to participate in the conference. The clinical faculty, prospective case presenter, and anthropologists formed a team to hold the conference. Next, the prospective presenter was asked to list several patient cases. To retrieve a wide range of suitable cases for analysis with an anthropological perspective, several topics were used by the case presenters (Fig. 1). They were also asked to consider including “probing questions” that they would like to discuss at the conference. Based on the request, the prospective presenters gave summaries of their patients’ cases.

Second, the anthropologists assessed and selected one or two cases. The criteria used to select potentially suitable cases for the conference are shown in Fig. 1.

Third, after choosing the cases for inclusion in the conference, the medical anthropologists and case presenters collaboratively constructed and elaborated the case presentation. This co-constructive interaction is a unique feature in the case conference model. Here, the anthropologists analyzed the preliminary written case and requested that the presenter retrieve additional contextual information deemed necessary for discussion and anthropological understanding. In some instances, the anthropologists suggested modifying or changing the “probing questions” formulated by the case presenters when they found that the questions might not promote an understanding of the case. Based on these comments, the case presenters rewrote and elaborated on the case. During this phase, the case presenters reviewed the chart, reflected on the cases and their performance, and sometimes conducted an additional interview of the healthcare professionals involved.

Finally, the anthropologists prepared their comments based on the elaborated cases. The anthropologists described several lessons that they learned after reflecting on the project. First, the comments are informative when they provide a theory that helps participants make sense of the conundrum, or form a question that can potentially reframe the clinicians’ perspectives on the case. In other words, the comments do not always have to answer the “probing question.” Second, the comments help clinicians follow the analysis of the anthropologists when they are explicit about the way they connect anthropological theories with the cases. For example, it is effective to quote particular phrases in the written cases since clinicians tend to regard the written case as authentic. Similarly, clinicians can better understand the case when anthropologists clarify the kind of phenomena they elicit from the quoted data before providing theoretical accounts. While introducing the technical terms or concepts from SBS, it is often imperative to identify which are technical to avoid confusing the clinicians since some technical SBS terms mimic lay terms (e.g., symbol, culture, exchange). Finally, preparing distinct comments from two or more anthropologists is preferred, if possible. This is because having a variety of perspectives on the cases exemplifies the multifaceted way in which social scientists analyze daily phenomena.

The implementation phase is approximately a 2–3-h session during which one case is discussed. First, the goal of the conference, which is to experience the clinical relevance of SBS through a discussion of real clinical cases, is explained. Next, a brief introduction of social and medical anthropology including contents related to (a) methodological approaches, for example, ethnography and participant observation; (b) anthropological theories, for example, cultural relativism, social construction of illness, etc.; and (c) concepts such as explanatory models, is given before the case presenter describes the elaborated case with “probing questions.” This is followed by a small group discussion by the participants and comments on the cases by medical anthropologists. If time permits, a plenary session is again encouraged, based on the anthropologists’ comments. Finally, the case presenter reflects on the entire process to conclude the conference.

### Development of the collaborative clinical case conference

**Fig. 3:**
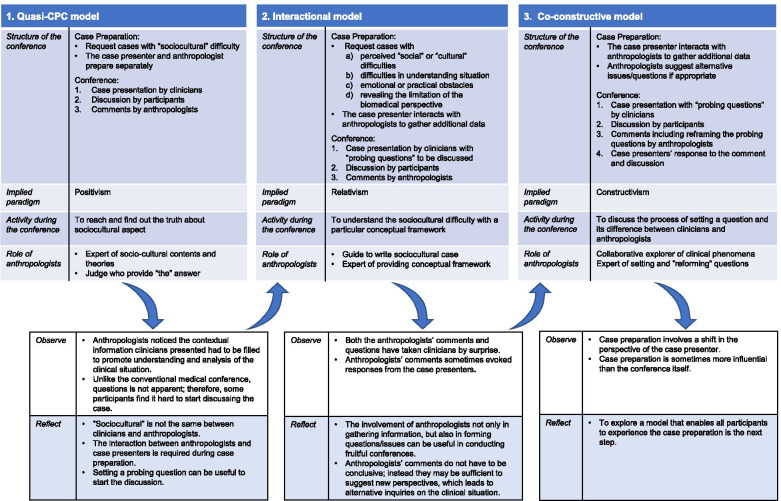
Evolution of the CCCC model and the concomitant action research process

The process of developing the CCCC can be summarized in three phases: quasi-CPC, interactive, and constructive phase. Each phase represents a distinct model and its features. The differences between the three phases and the processes by which we developed them are summarized in Fig. 3. Here, we simplified the gradual process of the development into three distinct phases to clarify the differences.

### Quasi-CPC phase

We initiated the conference with a structure almost identical to that of the conventional CPC. One significant modification to the structure was assigning two anthropologists as commentators, a decision aimed at demonstrating a variety of perspectives to understand the case, whereas medical diagnosis is usually a process to find one definitive answer.

From the evaluation of the “quasi-CPC” phase, the questionnaire found that most participants acknowledged the significance of learning SBS from their clinical cases. They generally perceived the anthropologists’ comments as valuable as they comprehensibly described the implicit aspects of the practice or patients’ situation. Notably, some participants appreciated the legitimate opportunity to discuss sociocultural issues that rarely become central in their workplace. However, some areas for improvement were suggested. First, the anthropologists pointed out that the information in the case presentation was not always sufficient to promote a sociocultural understanding of the case. This is not only because participants did not have the information required for the analysis, but also because their limited understanding of the sociocultural aspect made it difficult to judge what information was significant. Second, some participants found it difficult to start the case discussion without any explicitly suggested discussion points. This would be because clinicians are used to conventional biomedical conferences in which questions such as, “What is the diagnosis?” or “What is your management?” are the departure point. Accordingly, we posit the following two points: 1) the interaction between anthropologists and clinicians while preparing cases is necessary to ensure that appropriate and sufficient information is included in the presentation; and 2) some “probing questions” might be useful to initiate the case discussion.

### Interactive phase

To ensure interaction before the conference, we modified the case preparation from an isolated process by the case presenters to a collaborative one with anthropologists in two main ways. First, we changed the method of asking prospective presenters to list the cases for the conference after initially suggesting that they select cases with perceived “sociocultural” difficulties. We added three topics based on the reflection that their understanding of “sociocultural” would be limited and might lead to a narrow selection of cases being brought into the conference (Fig. 3). We also asked them to add “probing questions” that they wanted to discuss in relation to the case summary. Second, after the case was presented to the anthropologists, they requested additional information, such as contextual aspects and perception of other health professions, to ensure that adequate contextual information from an anthropological perspective is included in the case presentation.

The modifications done to ensure interaction before the conference had several effects. First, the anthropologists’ requests urged the clinicians to review their chart or talk to their colleagues about the case. This led them to become more cognizant of the difference between their perspectives and those of others and to rewrite the case presentation. Second, comments from anthropologists did not always provide a straightforward answer to the “probing questions” from the case presenters. Instead, anthropologists sometimes pointed out certain characteristics of the medical perspective by analyzing the way that the “probing questions” were formed, and proposed an alternative question through their anthropological analysis. For example, regarding a case involving a depressive elderly woman who “refused” care from physicians and other professionals such as, pharmaceutical therapy and home visits to her husband with dementia, the participating health professionals discussed how to overcome her rejection, whereas an anthropologist posed the question: “What was the lady protecting from the healthcare professionals?” Participants and clinicians in our team were impressed by the reformed questions of the anthropologists since it led participants to shift their perspectives on the case and the case presenters to remember otherwise forgotten information. A participant described the physicians’ perspectives as “interventionist,” which makes it challenging to understand patients’ worldviews. During the discussion, some participants noticed that physicians tend to assume that they are being neutral and exclude themselves from the case presentation and discussion. In the evaluative questionnaire after the trial of the interactive structure, one participant noted that, “since medical professionals cannot be objective no matter how they strive to be, they should be sensitive to their own filter.” This recognition of the unattainable nature of objectivity and the significance of being cognizant of the uniqueness of physicians’ perspectives shows an awareness of epistemology. This seems to be partly inspired by the explanation of participatory observation in the introductory lecture, but mainly by anthropologists’ attitudes towards discussing the positionality and perspectives of case presenters during the conference.

Two points were drawn based on these observations. First, anthropologists’ comments do not always have to be conclusive and close off the discussion. Instead, their essential role in the conference is to reframe the questions. We hypothesized that the conference is focused not only on the process of looking for an answer to the predefined problem, but also on that of posing a useful question that leads to a subsequent exploration of appropriate management. Second, allowing the case presenter to respond to the anthropologists’ comments might be useful since it would highlight how the anthropologists’ reframing could influence the clinicians’ understanding of the case and possibly lead to alternative actions within the case. To achieve this, we attempted to focus more on posing questions and exploring methods to secure the iterative process as much as possible between the case presenters and anthropologists in the preparation phase.

### Co-constructive phase

Given the reflection, we modified the structure in three ways. First, in the preparation phase, anthropologists guided the case presenter on what additional information to gather, and how to frame questions. Second, clinicians asked the anthropologists to clarify how they reframe the clinician’s questions when they comment on the case in the implementation phase. This is achieved by one anthropologist directly commenting on the case, and another explaining the premise and underlying perspective of the comment. Finally, we gave case presenters a chance to reflect on the discussion process after the anthropologists’ comments, to share the impact of the conference on their particular understanding.

Throughout the trials of the co-constructive structure, which is identical to the final structure of the CCCC, one notable finding was that the case preparation process provided a unique learning opportunity for the case presenters. Based on the influence of the anthropologists, case presenters were urged to review their charts, interview their colleagues with questions that clinicians rarely ask during work, and re-examine their perspectives on the clinical situation. Through this experience, some clinicians noticed that the understanding of particular clinical phenomena is not uniform, but differs among the involved healthcare professions (as an example of a case presenter’s learning, see Fig. 2). The anthropologists’ expertise in ethnography helped facilitate this process since they were able to notice which contextual information was missing and the kind of requests or questions that would be informative for the case writers to further their analysis. Therefore, the case preparation in this phase could be understood as a process of collaborative clinical case writing by case presenters and anthropologists, in which the former can perform a brief quasi-ethnographic exploration and experience a method of how social scientists explore “clinical” phenomena, and the latter can participate in the process of constructing the clinical reality. Here, anthropologists played the role of the collaborative explorer of clinical phenomena by (re)framing clinicians’ questions and guiding the exploration.

The iterative structure of the implementation phase enables a growing understanding of the clinical case through interactions among participants, case presenters, and anthropologists. Here, the case conference is not a deductive act for testing a hypothesis, but rather an explorative act to co-construct the understanding of the case. The co-constructive relationship between anthropologists and case presenters is a strength of this structure, which could lead to clinically relevant SBS learning experiences.

### Role stratification and learning of faculty clinicians and anthropologists: the CCCC as faculty development

**Fig. 4:**
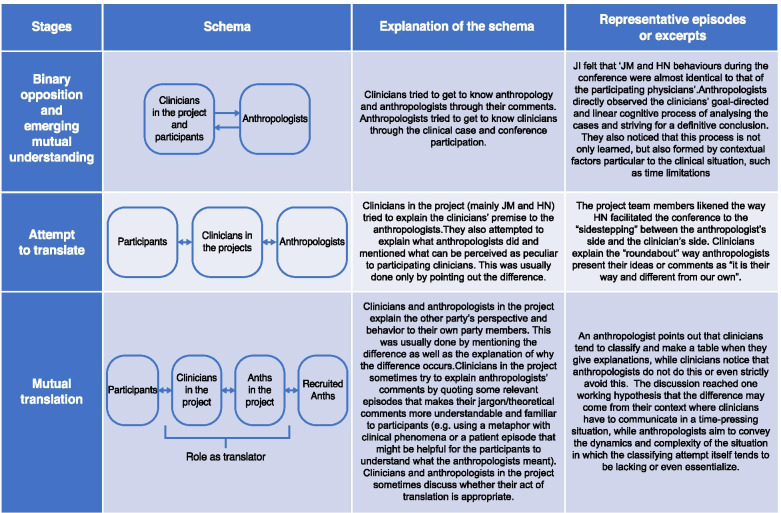
The stratification of the roles of clinicians and anthropologists during the CCCC development

In addition to the gradual change in the anthropologists’ role throughout the conference, the interaction of clinicians and anthropologists was gradually stratified during the model development, as schematically illustrated in Fig. 4. At the beginning of the project, clinicians described their clinical contexts to the anthropologists, and anthropologists explained the characteristics of their disciplines in the preparation and implementation of the conference. The conference became a place where a group of clinicians and anthropologists could gather and explicitly reveal their reasoning processes. As a result, a degree of mutual understanding between faculty clinicians and anthropologists emerged. Participants’ reactions to the anthropologists’ comments allowed the latter to know which particular theories and findings were complementary to the physicians’ perspectives and easy for them to understand. The anthropologists found it interesting that some scholarly “obsolete” theories were very relevant to the physicians, whereas other cutting-edge approaches were not. This understanding of the academic-clinical gap was a significant lesson for the anthropologists who participated in our project.

In the latter phase of the project, some clinicians and anthropologists came to play a “translator” role (Fig. 4). For example, experienced anthropologists in the conference guided more novice colleagues by giving tips such as, “take care of the academic-clinical gap.” These translational attempts were particularly influential for “novice” anthropologists who were entirely alien to medical education since such attempts functioned as “scaffolding” to promote their participation in and learning of medical education.

The clinicians in the research team (JM and HN) learned how the stances of the anthropologists were different from those of the clinicians. While clinicians tend to assume the cases *are* patients and their diseases, anthropologists tend to recognize that they are sediments of the process between the presenting clinicians and their contexts. This gap led to different targets of analysis during the case conference. In particular, clinicians try to know the patients and their health problems, whereas anthropologists go beyond these and include the relationship between case presenters, patients, other stakeholders, and even the perspectives of the case presenters, as well as the conference participants. In other words, while the task for clinicians during the conference is an analysis *of* the case, the task for anthropologists is an analysis *through* the case. The presented cases function as an epistemology for anthropologists to understand the clinicians and their perspectives, which includes their clinical practice. Such comparative understanding helped the clinicians explain how their perspectives differ from those of the anthropologists during the conference. This growing mutual understanding was an additional, but significant, process accompanying our research project.

## Discussion and conclusion

The CCCC developed in the study successfully contributed to promoting participants’ recognition of the clinical significance of SBS. The unique features of this model were the collaborative writing of clinical experience during the preparation phase and the iterative structure during the implementation phase. In particular, the former seems to provide case presenters with a profound, unique learning opportunity. They can revisit their clinical experience through the way that social scientists explore clinical phenomena. Although case-based learning has frequently been reported as a teaching method to promote the integration of clinical medicine and non-clinical disciplines [18], the involvement of scholars from other disciplines in the *writing* of clinical experiences is scarcely reported on. In the CCCC, this involvement seems to promote the integration of clinical medicine with the contents, theories, and research methods of SBS.

Reflecting on the whole process of model development, the transition from the conventional case conference to our final co-constructive model may be understood as one that involves a shift in underlying assumptions about reality [27, 28] from a biomedical to a SBS paradigm (Fig. 3).

The paradigm of the former is positivism, and the goal of the conference was to verify the clinicians’ diagnostic hypothesis with a definite answer (diagnosis) and explore the clinicopathological correlation. Clinicians prepare the material to propose their diagnostic hypothesis and establish its validity through deductive reasoning. Pathologists approach the case with established, controlled experimental pathological methods. Although each part interacts before and during the conference, the basic premise is that a definitive, stable truth exists, despite the approach clinicians and pathologists take. The case is regarded as a separate entity from the presenters and participants.

In contrast, a dominant paradigm behind SBS is social constructionism, in which knowledge is constructed based on the interactions between people [29]. The CCCC represents this paradigm since the whole process of the conference, including case preparation, focuses on the exchange between each party (SBS scholars and clinicians) and how they view the case. This point is also reflected in the findings from the evaluative questionnaire, which show that some participants noticed the characteristics of clinicians’ perspectives during the conference.

As a supplemental analysis, we delineated the process of the role stratification of clinical faculty and anthropologists, accompanied by their evolving mutual understanding. For medical anthropologists, this project offered a legitimate opportunity to participate in writing authentic clinical cases. They elaborated on the practical tips regarding the collaboration with clinicians, which functioned as the “scaffolding” for novice anthropologists. The clinicians in the project team discerned their particular strengths and played a “translator” role in the conference. On this point, it is plausible to regard the research project as a community of practice providing “an important venue for faculty development (FD)” for both SBS scholars and clinicians who were not familiar with teaching SBS in medical education [30]. However, we expanded the discussion to articulate the facilitation of collaboration across disciplines by applying the “landscape of practice (LoP)” [31].

The LoP emphasizes the dynamic process of how more than two communities of practice can interact. For a productive interaction, the role of “brokers” in negotiating the exchange of knowledge between the CoPs is emphasized as a key; such “brokers” form “knowledgeability” through which they establish complex relationships with respect to an LoP and are recognized as reliable, legitimate practitioners in the LoP [31]. In our project, the conference became an LoP where two CoPs (clinicians, including clinical faculty, and anthropologists) intersect. The “translator” could be understood as the emergence of the broker role that productively influenced the collaboration. For instance, the series of lessons derived from the “translator” perspective, such as the “academic-clinical gap,” would be an LoP-specific knowledge, promoting novice anthropologists to develop knowledgeability and become effective teachers. Another notable point was that the first “translator” (HN) in our team were medical education researchers who were familiar with various learning theories and qualitative research methodologies originating from different paradigms [32]. The prior experience of switching between different paradigms might make it easier to develop knowledgeability and play a broker role in the early phase of a project. This point would also be applied to anthropologists who are trained at being aware of their own assumptions as well as those of others.

This study has several potential limitations. First, we only retrieved data on the perceptions of the case presenters and conference participants. Further attempts with more data are necessary to describe outcomes, such as participants’ behavioral change or its influence on patient care and health outcomes. Second, the CCCC was developed only through our teaching for family physicians and medical students. Further studies are required to verify its usefulness and effect in other medical contexts. Third, our study only worked collaboratively with one discipline of SBS, which is anthropology. While we expect that it also applies to sociology and psychology, as there are many similarities among these fields, further exploration is required. Fourth, the CCCC was mainly developed in the context of voluntary sessions for participants. Therefore, it is necessary to check whether this model works in the context of a compulsory curriculum. Finally, the CCCC will not suffice for effective SBS teaching and should be coupled with institutional support and curriculum change since it is suggested that effective SBS teaching also requires alleviation of structural and curriculum barriers in medical school [4, 33].

Despite these limitations, several implications can be drawn from this study. We propose that medical teachers who aim to integrate other disciplines into clinical medicine education should attempt to more rigorously involve scholars from other disciplines into students’ writing of clinical experience. The CCCC provides a concrete educational opportunity where medical students and clinicians can interact with SBS scholars and integrate SBS into clinical experience across undergraduate, postgraduate, and CPD. Such opportunities could lead to the development of clinicians and medical teachers who can acknowledge and elaborate on the value of SBS, which can counter an underlying deep belief discrediting the value of SBS. Moreover, our model might be applicable to other non-SBS disciplines—for example, basic medical sciences. Although our model was developed through experiences with anthropologists who are experts in writing about authentic phenomena, we expect that an exploration of collaborative writing with scholars from other disciplines might be fruitful as well. By exploring the learning that occurred through the writing cases under the guidance of a range of scholars, including both SBS and basic sciences scholars, and the features of the process with which such learning is promoted, medical educators can develop better teaching strategies to provide integrative learning for medical students and physicians in clinical contexts.

Multiple paradigms may be applied to the role of clinical cases when designing teaching methods. The patients’ cases are not simply finished materials for discussing and justifying diagnosis and management, but rather an unfinished activity subject to social construction. From this perspective, the act of writing and discussing a case under the guidance of anthropologists can be understood as a process of co-constructing what is clinically relevant. Thus, SBS scholars in medical education would have the significant role of more closely participating in the construction process and attempting to reconstruct their perspective on what is called “clinical relevance.” Such reconstruction attempts may also help clinicians and medical students to recognize the importance of topics (e.g., societal forces on individual behavior) that have been considered clinically irrelevant or simply not taken into account. For clinical faculty, it is important to invite SBS scholars into the clinical conversation, including case conferences, to rigorously connect clinical practice and SBS contents and ensure such learning. It is suggested that such learning is also necessary for clinical teachers since they have to guide the integration during educational sessions. For example, problem-based learning (PBL) tutors have to learn how to integrate SBS into PBL sessions for effective SBS learning [6].

We do not intend to suggest that one model fits all in teaching SBS since each paradigm has its particular strength. Instead, we suggest that faculty who plan on teaching SBS via clinical cases should be cognizant of which models they intend to apply since the structure and role of SBS scholars should vary based on the model. For example, for students lacking in clinical experience and knowledge of SBS contents, it might be better to structure a conference similar to the quasi-CPC phase. However, clinicians or medical students with clinical experience would benefit more from the CCCC than a quasi-CPC structure since the former is more relevant to their real experience and involves an awareness of their implicit perspective. Therefore, it is beneficial for both clinical faculty and SBS scholars to flexibly navigate between varying paradigms to tailor the conference structure according to the educational goal and maximize the effectiveness of teaching SBS in medical education.

Our analysis on the collaborative process suggests that the structure of CCCC functioned as faculty development which contributes to the generation of practical teaching tips as well as mutual understanding between clinicians and anthropologists if only effective facilitators are prepared to function as “brokers.” Recent work reviewing anthropology in medical education points out that some anthropologists are unexpectedly involved in and, hence, not well-prepared for medical education; this calls for training of novice anthropologists in medical education [33]. The CCCC model would provide a practical model for novice anthropologists and other SBS scholars to learn not only biomedical terminology but also how clinicians actually go through their cases and how they may work on their perspectives to generate effective learning.

Our findings and analysis regarding the process of collaboration provide insight into future research on FD and SBS teaching. In the context of the integration of different disciplines, the LoP illuminates the significant characteristics of the collaboration and the role and competency of faculty. For example, we consider the “broker” role to be a potentially important feature of faculty for the successful integration of SBS in medical education. Another point that deserves attention is the potential of medical education researchers with experience of collaboration who can move flexibly across different paradigms and play the “translator” role. Regarding this point, research in medical education has also been shown to involve collaboration between researchers with different paradigmatic orientations, which can potentially contribute to the productivity and effectiveness of medical education. However, to maximize the generative aspect of the collaboration or “the multidisciplinary edge effect,” it is suggested that an understanding of one’s own paradigm and those of others is imperative [27]. We argue that the same issue is pertinent to teaching SBS in medical education. It requires collaboration between SBS scholars and clinicians, and opens up a space that potentially entails “the multidisciplinary edge effect.” Thus, the FD for SBS educators in medical education*—*whether they are clinicians or SBS scholars*—*should involve collaborating with others who have different paradigmatic orientations. Such collaborations may be fueled by sharing lessons and experiences from multidisciplinary collaborations in medical education research. We believe that such contributions improve the potential productivity and effectiveness of teaching SBS in medical education.

In conclusion, when medical teachers integrate SBS into clinical medicine, they should be cognizant of the paradigmatic difference between biomedicine and SBS and strive to construct an epistemological bridge across the divide. Strategies for teaching SBS, as well as future FD in SBS in medical education, should focus on the collaboration between faculty with different paradigmatic orientations.

## Supplementary Information

**Additional file 1: Supplementary material 1.** Topics and comments in undergraduate conferences.

**Additional file 2: Supplementary material 2.** Topics and comments in postgraduate and CPD conferences.

**Additional file 3: Supplementary material 3.** Raw data of questionnaires

## Data Availability

The datasets supporting the conclusions of this article are included within the article and its additional file.

## Abbreviations

CCCC: Collaborative clinical case conference model
CPC: Clinicopathological Conference
CPD: Continuous professional development
FD: Faculty development
LoP: Landscape of practice
PBL: Problem-based learning
SBS: Social and behavioral sciences
