# Supplementary material for: A collaborative clinical case conference model for teaching social and behavioral science in medicine: an action research study
Source: BMC Med Educ. 2021 Nov 12;21:574. doi: 10.1186/s12909-021-03009-8 (PMC8590366; doi:10.1186/s12909-021-03009-8)
Supplement: Supplementary file 1 — Additional file 1: Supplementary material 1. Topics and comments in undergraduate conferences. [file 12909_2021_3009_MOESM1_ESM.pdf]

A Collaborative Clinical Case Conference Model for Teaching Social and Behavioral Science in Medicine: An Action Research Study

Junichiro Miyachi[abe*], Junko Iida[c], Yosuke Shimazono[d], Hiroshi Nishigori[ae]

[a] *Center for Medical Education, Graduate School of Medicine, Nagoya University, Aichi, Japan*

[b] *Hokkaido Centre for Family Medicine, Hokkaido, Japan*

[c] *Faculty of Health and Welfare, Kawasaki University of Medical Welfare, Okayama, Japan*

[d] *Center for Global Initiatives, Osaka University, Osaka, Japan*

[e] *Medical Education Center, Graduate School of Medicine, Kyoto University, Kyoto, Japan*

***Corresponding author:**

Junichiro Miyachi

65 Tsurumai-cho Showa-ku Nagoya, 466-8560, Aichi, Japan

Tel: +81-52-744-2997

Fax: +81-52-744-2644

E-mail: j.miyachi@hcfm.jp

Supplementary material 1. Topics and comments in undergraduate conferences

Date	No.	Case summary topics	Comment summary	Number of teachers and participants		
				CT	MA	Students
8-Jan-15	1	Why is the perception of disease (ex. Cancer, AIDS) different across people and countries?	Impact of social, cultural, political, and economical situations on perceptions of sickness and life	2	1	10
	2	Decision-making on terminal care when conflict exists between opinions of stakeholders (doctor, patient, patients' families)	1. Importance of collaborative decision-making, rather than informed "consent"<br>2. The knowledge of physicians is not superior to, but different from that of patients' (cultural relativism)			
	3	Decision-making on the care of a patient with dementia who has no relatives	Non-kinship relationship involving a solitary person and its potential in decision-making			
30-Oct-15	4	Management of or communication with a lady with uterine cancer who wants a baby	Narrative	2	3	10
	5	How to manage an autistic child who tries to enter a highway because of his obsession with cars and his mother	Dominant story and alternative story			
	6	ABCs of communication with hospitalized patients for cancer treatment	1. Doctor-patient relationship<br>2. Transition of a framework in a dialog			
1-Jul-16	7	An experience where the student could not translate the patient's word "dizzy" into medical terms	Daily-life words and medical terms; Connotation and denotation; Illness narratives	3	2	10
	8	Discrepancy between self-image and objective impression in video review	ABCs of participatory observation, practice of observation, physical exam, and doctor-patient relationship			
27-Oct-16	9	Management of a patient with multi-organ cancer metastasis (The validity of informed consent and whether the patient is satisfied with the decision)	Narrative	2	2	10
	10	Management of an autistic patient who calls an ambulance very often	Cultural/medical Anthropology and its perspective			
17-Oct-17	11	Is a medical student a "health professional?" If so, to what extent?	Legitimate peripheral participation	2	2	30
	12	The validity of care for a patient with terminal lung cancer who wanted to return home, but healthcare professionals could not guarantee the possibility of allowing him to do so	Contrast between logic of choice and logic of care	2	2	30
28-Jul-18	13	How should we communicate with a patient who laments an adverse event (stroke) after a prospective surgical procedure for cranial aneurysm?	Disease vs illness, explanatory model, illness as a constriction of the lived world and its care	7	3	98
16-Oct-18	14	Concern about the decision-making of a patient was dependent on the emotions of the patient and his family members rather than on "rational scientific" evidence	Differences between practical knowledge in the clinical arena and scientific knowledge	2	2	30
	15	How can we know the influence of the disease on the life of a patient and his/her family members?	Understanding the illness experience as an unfinished story (subjunctivizing one's illness narrative)	2	2	30

Note. Adapted from "Managing uncertainty: Collaborative Clinical Case Conferences for physicians and anthropologists in Japan," by Iida, J. and Nishigori, H., in I.L. Martinez and D.W. Wiedman (Eds.), Anthropology in Medical Education: Sustaining Engagement and Impact (p. 81–82), 2021, Cham: Springer Nature Switzerland AG. Copyright © 2021, Springer Nature Switzerland AG. Adapted with permission.
CT: Clinical Teacher, MA: Medical Anthropologist
