# Supplementary material for: A collaborative clinical case conference model for teaching social and behavioral science in medicine: an action research study
Source: BMC Med Educ. 2021 Nov 12;21:574. doi: 10.1186/s12909-021-03009-8 (PMC8590366; doi:10.1186/s12909-021-03009-8)
Supplement: Supplementary file 2 — Additional file 2: Supplementary material 2. Topics and comments in postgraduate and CPD conferences. [file 12909_2021_3009_MOESM2_ESM.pdf]

A Collaborative Clinical Case Conference Model for Teaching Social and Behavioral Science in Medicine: An Action Research Study

Junichiro Miyachi[abe*], Junko Iida[c], Yosuke Shimazono[d], Hiroshi Nishigori[ae]

[a] *Center for Medical Education, Graduate School of Medicine, Nagoya University, Aichi, Japan*

[b] *Hokkaido Centre for Family Medicine, Hokkaido, Japan*

[c] *Faculty of Health and Welfare, Kawasaki University of Medical Welfare, Okayama, Japan*

[d] *Center for Global Initiatives, Osaka University, Osaka, Japan*

[e] *Medical Education Center, Graduate School of Medicine, Kyoto University, Kyoto, Japan*

***Corresponding author:**

Junichiro Miyachi

65 Tsurumai-cho Showa-ku Nagoya, 466-8560, Aichi, Japan

Tel: +81-52-744-2997

Fax: +81-52-744-2644

E-mail: j.miyachi@hcfm.jp

Supplementary material 2. Topics and comments in postgraduate and CPD conferences

Date	No.	Case summary topics	Comment summary	Number of teachers and participants		
				CT	MA	Participants
12-Jun-15	1	Terminal care of a dying priest who is also worthy in the community	1. Friction and rivalry between social roles and personal life<br>2. Transition of death and dying among eras (Aries) in Japan and process of dying	4	6	30
	2	Management of a socially isolated family with a muscle and intelligence disorder who is totally separated from the world of modern medicine	1. Ethno-graphic description of the case<br>2. Recovery of patient autonomy			
8-Nov-15	3	A patient who became happy with a stoma and the presenter's surprise with it	1. Life history<br>2. Inconsistency between one's words and actions, asymmetry between doctors and patients<br>3. Pollution, taboo, and order	5	7	40
	4	A 100-year-old woman whose family members prioritize religious routines and rites over their mother's care	1. Cultural competence<br>2. Overview of newly risen religions, secularism, and “power of *imo* (female)”<br>3. Gap between a family's ideology and situation			
10-Feb-16	5	An elderly “garbage” house and her decent family	Relationship between the family and the local community; the house and history of the family	3	2	5
	6	An old man with dementia and his wife with depression who reject prescription and care	Female position and role in a Japanese family; system theory thinking			
10-Jun-16	7	How do we manage a problem with a patient who denies medical care?<br>(Same case as no.6)	1. Family history and social relationship (role as a mother and role as a wife)<br>2. Rephrase the question to “What did this old man try to guard from health professionals?”	4	6	33
6-Nov-16	8	Management of a woman with SLE who has a university second opinion outpatient service	1. Patient's logic and doctor's logic<br>2. Narrative, its structure, and the second opinion service as a place where patients explore their narratives	5	5	40
12-May-17	9	The goal and extent of medical home care for a deteriorating iNPH patient	1. Doctor-patient relationship from the perspective of gift theory.<br>2. Physical and social experience of “I cannot” and care to elicit novel “I can”	5	5	29
22-Jul-17	10	Terminally-ill patient with COPD who insisted on toileting alone. A dilemma between autonomy and care.	A transformation of a status around receiving an aid for toileting, medication and self-perception, multiplicity of living ways in terms of choices in managing their daily activities	3	5	18
12-Nov-17	11	A patient with adrenal insufficiency treated with corticosteroid, whose pharmacist daughter misunderstood the disease pledged to use supplements. How to manage a conflict of opinion?	Co-existence of knowledge of treatment from different medical sectors (professional and folk sectors);<br>The (un)acceptance of the death of the daughter’s mother, subjunctivizing illness narrative	3	7	30
27-Jan-18	12	A patient with CKD who attributes a range of symptoms to medications and rejects dialysis because of his concern that dialysis will become a burden on his family	Relational autonomy, local biology, social body, and a sense of having boundaries aggressed by transfusion, dialysis, and medication	2	4	21
24-Feb-18	13	A patient admitted with diabetic nephropathy exacerbation caused by interruption of medication who attempted to send a gift to a resident to thank her for her care	The conflation of adherence with intelligence, a classical theoretical concept of gift and exchange	7	5	60

Note. Adapted from “Managing uncertainty: Collaborative Clinical Case Conferences for physicians and anthropologists in Japan,” by Iida, J. and Nishigori, H., in I.L. Martinez and D.W. Wiedman (Eds.), Anthropology in Medical Education: Sustaining Engagement and Impact (p. 81–82), 2021, Cham: Springer Nature Switzerland AG. Copyright © 2021, Springer Nature Switzerland AG. Adapted with permission.
CT: Clinical Teacher, MA: Medical Anthropologist
