# Supplementary material for: A collaborative clinical case conference model for teaching social and behavioral science in medicine: an action research study
Source: BMC Med Educ. 2021 Nov 12;21:574. doi: 10.1186/s12909-021-03009-8 (PMC8590366; doi:10.1186/s12909-021-03009-8)
Supplement: Supplementary file 3 — Additional file 3: Supplementary material 3. Raw data of questionnaires [file 12909_2021_3009_MOESM3_ESM.pdf]

# A Collaborative Clinical Case Conference Model for Teaching Social and Behavioral Science in Medicine: An Action Research Study

Junichiro Miyachi[abe*], Junko Iida[c], Yosuke Shimazono[d], Hiroshi Nishigori[ae]

[a]*Center for Medical Education, Graduate School of Medicine, Nagoya University, Aichi, Japan*

[b]*Hokkaido Centre for Family Medicine, Hokkaido, Japan*

[c]*Faculty of Health and Welfare, Kawasaki University of Medical Welfare, Okayama, Japan*

[d]*Center for Global Initiatives, Osaka University, Osaka, Japan*

[e]*Medical Education Center, Graduate School of Medicine, Kyoto University, Kyoto, Japan*

***Corresponding author:**

Junichiro Miyachi

65 Tsurumai-cho Showa-ku Nagoya, 466-8560, Aichi, Japan

Tel: +81-52-744-2997

Fax: +81-52-744-2644

E-mail: j.miyachi@hcfm.jp

Supplementary material2: Raw data of questionnaires

# June, 2015

| Profile | Summary of Learning and point of improvement |
|---|---|
| MD in a clinic and a university hospital | I noticed an affinity between PCCM and anthropological view |
| MD in a community hospital | The world of anthropology is not far from what physicians think about during daily work<br>Doctors tend to intervene something |
| MD and director of a private clinic | Significance of different perspectives<br>Acknowledgement of diversity is important |
| Medical Student | I wanted to go beyond qualitative research and learn a discipline which put a range of phenomenon into concrete words. I enjoyed listening to the MC. |
| MD in a university | It was good to be able to formulate non-biological questions and discuss them openly. |
| Not stated | It gave me a lot of words to frame my habit of doing things vaguely. I felt like I was given a way to deepen my understanding of the importance of participation.<br>The difficulty of participant observation. Now I want to intervene as a medical professional. I realized that I am struggling to fit into the framework of medicalization. I realized that I don't have enough time to understand others ethnographically. |
| MD in a university | It was good to be able to understand the thoughts of the attending physician based on actual cases, and to hear in depth from a medical anthropologist in another position.<br>I realized new ways of describing non-medical information when writing medical records. |
| Medical Student | I participated because I wanted to know how medical anthropology is discussed in real situations.<br>There were some things I felt about my "role" as a clinical doctor, such as to what extent medical professionals should intervene, and whether it is meaningful to simply write down daily field notes. |
| MD in a community hospital | It was interesting to discuss things that I thought were natural in terms of social and medical conventions (common sense).<br>I hope that medical care will become a place where people can seriously think about things that they |

| | |
|---|---|
| | couldn't get deeply into before (especially things that they would forget if they were left as they are). |
| MD in a university | I was interested in anthropology.<br>I realized that it is difficult to understand others!<br>The meaning of participation and the meaning of doctors being in the community became somewhat clearer. |
| MD in a university | I wanted to know about the difference in perspective between family physicians and medical anthropologists.<br>I wanted to know about the difference in perspective between family physicians and medical anthropologists. ・Medical professionals cannot easily escape the tendency to cut out certain parts of patients' and families' lives, identify problems, and try to solve them within the framework and approach of medical professionals.<br>Conflict with the fact that their solutions and judgments will be evaluated (will everyone think that it is the right decision to leave the problems as they are?) |
| Not stated | The case conference was very interesting. |
| MD in a community hospital | I had been discouraged because I thought that my interest could not be solved in the medical field (and because others told me that it was not something that doctors should do), but I felt that there was a way. |
| Psychologist | I am a psychology major and interested in family medicine, so I wanted to know what kind of study "medical anthropology" is and how it collaborates with family medicine.<br>I wondered if it would be okay for a psychologist to do this. |
| MD in a university | I wanted to find out how the perspective of anthropology can be applied to our clinical practice.<br>I was able to recognize once again that there is no single answer, and that by carefully describing our awareness, we can gradually come to see it. |
| Not stated | I participated without knowing anything about it.<br>The opinions of anthropologists are frustrating for those of us in the medical field because there is no |

| | |
|---|---|
| | answer (no connection).<br>Participant observation and community medicine: I rediscovered myself again by listening to the ethnographic collaboration. |
| MD in a university | I was able to think deeply. I gained a lot of perspectives. I recognized the limitations of knowing others. |
| MD in a hospital | In advance...<br>How to apply medical anthropology to clinical practice<br>How to apply the perspective of medical anthropology to clinical practice<br>How can I apply the perspective of medical anthropology to my clinical practice?<br>I found Dr. U's theory of "ethnographic beauty" interesting.<br>How to apply the perspective of medical anthropology to clinical practice<br>Two complex and generalizable cases were presented! The fact that there were |

## November 2015

| Profile | Comments |
|---|---|
| MD in a university hospital | I was a little uncomfortable with the presentation that seemed to dare to play up the stereotype of family medicine. I wondered if it was to highlight the difference from cultural anthropology and the subsequent presentation. |
| Not stated | I have read Arthur Clyman's book and was interested in it.<br>It was interesting to see the reasons and backgrounds of the people who asked questions in the Q&A session, because I felt they had their own medical backgrounds.<br>I felt that I should be sensitive to the filter of myself, because even if I try to look at a patient or person objectively, a medical professional cannot be objective. |
| Other | I wanted to increase my knowledge of how to reconcile the perspectives of the medical staff and those of the patients when I go to the homes of patients with different lifestyles and cultures. |
| MD in a clinic and a hospital | I was interested in medical anthropology because I heard a lecture on it about 20 years ago. |
| MD in a hospital | I don't know what medical anthropology is, but I thought it smelled like the humanities. I am interested in narrative medicine, and it seemed to be related.<br>I was able to come into contact with the perspective of an anthropologist. I was exposed to the viewpoint of an anthropologist, which I would never have thought of. |
| Not stated | There are academic categories for what I have been asking patients. |
| MD in a hospital | I was surprised to know that my own medical treatment is also a participatory observation... |
| Not stated | I have learned how to overcome relativism in narrative medicine |
| MD in a clinic | I felt that some of the theories of family medicine can be supported by anthropology.<br>I felt that what the medical anthropologist talked about included what I value in family medicine and my own practice, and that it might be possible to |

| | |
|---|---|
| | provide education from this. |
| MD in a hospital | I participated because I am studying the BPS model and thought I could learn more about it from anthropology. |
| MD in a clinic | I found the seeds of a medical anthropological perspective in what I usually think about, and that there is similarity with family medicine. |
| Not stated | As a former humanities major, I was interested in the opinions and thoughts of doctors and nurses. |
| MD in a hospital | I think I may be able to gain new perspectives and horizons through actual medical care. |
| MD in a clinic | That family medicine and medical anthropology may overlap in everyday clinical practice |
| MD in a hospital | I have always been interested in anthropology.<br>I don't get a chance to listen to anthropologists unless I have this opportunity. |
| MD in a clinic | I want to learn more about the patients.<br>I want to see how anthropologists conduct their interviews. |

June, 2016

| Profile | comments |
|---|---|
| MD in a clinic | I felt that it would be easier to get into the discussion if the evaluation and exclusion of organic diseases were included more before the social approach.<br>I felt that there were a lot of subjective approaches and arguments, and not enough discussions based on scientific evidence. |
| MD in a hospital | Medical professionals as observers and interveners should also be considered. That anthropologists are experts in "asking questions". I thought the atmosphere was like that of the Balint Group. |
| MD in a hospital | I was given an opinion from the perspective of a non-medical person, which made me think about whether the treatment we are giving is really good for the patient. I would like to have more sessions with many cases. (Cancer, disabled, mental illness, etc.) |
| Not stated | I got goose bumps from the flow of "protecting" and "shame" (2 other people were also thrilled) |
| MD in a clinic | I used to think that talking was the basis of health communication, but now I realize that there are some things that can no longer be protected by medical intervention or talking in the first place. |
| MD in a clinic | It was good to hear from multiple anthropologists. |
| MD in a hospital | "What was the old lady protecting? →I got goosebumps at the response that it might be "shame" that the old lady protected. I would like to utilize the concept of inside/outside in my interviews. |
| MD in a hospital | I was able to learn about various approaches in the discussion, which was helpful. I felt the time was too short. |
| MD in a hospital | I was able to get a concrete image of a case conference. It was a little difficult to understand the intention of the "questions" in the initial discussion. |
| MD in a hospital | I attended the seminar because I thought it would give me a new perspective on difficult cases. The discussion was very insightful and I would like to participate again. |
| MD in a hospital | It gave me a new perspective. |
| MD in a hospital | I was thrilled to hear "I think this person was protecting "shame. It was a new perspective. |
| Not stated | I was surprised by the idea that we (medical professionals) should be included in the systems theory. |

| | |
|---|---|
| Not stated | I was satisfied with the conclusion that medical anthropologists are "experts in questions. |
| MD in a hospital | I was able to gain a new lens on medical anthropology. |
| MD in a hospital | I notice the difficulty to understand culture. |
| MD in a hospital | I thought I could learn how to think about the possibility that some people unconsciously benefit from seemingly irrelevant, meaningless, or painful choices. |

November 2016

| Profile | Comments |
| --- | --- |
| MD in a hospital | Interest in anthropology itself<br>I did not understand anthropology itself yet. I wanted to know about the parts that have nothing to do with medicine. |
| MD in a clinic | While the participants were aware of the necessity and importance of conceptual teaching and listening, I felt that the OARS technique in the motivational interviewing method would be useful as a conversation and communication skill to elicit and concretize it. |
| MD in a hospital | I was hoping to learn about concepts that transcend the boundaries of conventional medical treatment, or to be able to verbalize what I feel in actual practice (motivation for participation). |
| MD in a hospital | I felt that everyone had many resources to draw from, which motivated me to study more.<br>I thought "I see what you mean" when you talked about logic... (followed by questions about logic and comments on the development of logic) |
| MD in a clinic | If nurses could be trained to act as interpreters, it would be possible to see the story to some extent in the examination room and save time.<br>Story of Chigusa no Mori<br>We have not yet been able to verbalize what we are doing, so we would be very happy if you could help us. |
| MD in a clinic | What I remembered from yesterday's lecture on VBP was further activated in today's review meeting. |
| MD in a hospital | I think it's good to be able to connect doctors to the whole process, but I can't think well because environmental factors (lack of time) come to mind first. |
| MD in a hospital | I would like to expand the scope of case conferences focusing on pathology and diseases. |
| MD in a clinic | I want to broaden the window for understanding patients.<br>Listening is not only a cathartic healing process for the other person, but also a process of rewriting the story and making it more acceptable by putting the events in a different context and |

| | |
|---|---|
| | giving them meaning through dialogue. |
| MD in a clinic | This is something that I usually think about (although I may just not be calm or understand it). |
| MD in a university hospital | I wanted to know how doctors/medical professionals and anthropologists can collaborate. |
| MD in a clinic | I felt that the lecture verbalized what I practice in my daily practice. |
| MD in a hospital | I participated in the workshop in the hope of gaining a deeper understanding of human beings through medical anthropology.<br>I learned how to express the situation of patients in a literary way (difficulty of verbalization), and I thought this would help me understand patients better. |
| MD in a university hospital | I originally entered medical school because of my interest in medical anthropology. I was glad that there was a place like this where my interests were emphasized.<br>The facilitator's comments on the group discussion were appropriate and good. |
| Not stated | I thought that exploring the logic of the patient's life world and understanding the differences and similarities between the positions of the patients in order to understand the reasons for their visits and their illnesses would broaden the range of medical care that can be provided to patients who visit the emergency room with the same complaints over and over again or who have difficulty understanding what they want to solve. |
| MD in a hospital | Because I have experienced many cases that are difficult to deal with only from a medical point of view, and I wanted to have an opportunity to break away from that and/or to improve a little.<br>I was relieved to know that other doctors also have similar problems (just like today's SLE patient).<br>I think it is easier to collaborate with primary care physicians (nurses and pharmacists as well), because I think they have a larger percentage of patients who are treated from a holistic perspective than specialists, not just individual diseases. Is it possible to increase the number of learning opportunities such as WS/seminars to deepen medical anthropological perspectives and ideas? |

| | |
|---|---|
| MD in a hospital | I thought that medical anthropology would be useful in dealing with the community.<br>Doctors have a habit of thinking in terms of setting outcomes/goals, don't they need to do so?<br>In medical care, we often "intervene" more forcefully than just participant observation (or rather, we think of it as an assumption). |
| Md in a clinic | I was originally interested in anthropology and wanted to learn how it could be applied to my daily practice.<br>I was able to deepen my understanding of anthropology by being able to discuss it with other members who have a similar awareness.<br>It would have been good to have more comments from the perspective of anthropology in the group discussion. |
| Clinic pharmacist | It was the first time for me to encounter the term "medical anthropology," and I had no idea how anthropology was connected to medicine... |
| MD in a hospital | I would like to learn more about examinations of foreigners, people with different cultures, and people who place importance on religion. |
| Pharmacist | I think the selection of cases could have been simpler.<br>I think the example case is a drug interaction. |
| MD in a hospital | I was attracted to the term "medical anthropology" when I applied. As a practitioner of community medicine, I thought I would be able to receive advice, suggestions, and ideas on methodology for medical treatment from an anthropological perspective. |
| Md in a clinic | Basics of a new stance in daily practice<br>I thought it would be good to use cases from different situations, i.e., daily outpatient cases (sometimes commonplace). |
| MD in a hospital | Isn't it a basic requirement for doctors to be narrative in the first place? Isn't the problem that there are more and more doctors (due to specialization) who can't do the obvious things like listening to people and considering their social background? If that is the case, I felt that presenting difficult keywords like |

| | |
|---|---|
| | "anthropology" would not reach the doctors who really need improvement. Personally, I learned a lot from the language that showed the points to be narrative. |
| MD in a hospital | The number of people and background to be discussed was appropriate. |
| MD in a hospital | I realized that when I am under a lot of pressure to solve a problem, I tend to think less about narratives and anthropological background. I may be afraid of becoming like "relieving the patient's suffering -> reinforcing the further deterioration of Dr. B? |

201705 Takamatsu

| Profile | Comments |
|---|---|
| MD in a hospital | Once again, when you change the persona from "the doctor" to "the patient," the way you perceive it is completely different. The key phrase "doctors become medicine" was obtained. |
| MD in a hospital | I liked the fact that I was able to deal with the social aspect of being physically "unable". |
| MD in a hospital | I was able to learn about concepts such as "gift exchange" and "contraction of able experience. |
| MD in a university hospital | It was interesting to read the actions of doctors from an anthropological perspective. Doctors are always thinking, "To what extent is this my job? I wonder why. I wonder why that is. |
| MD in a hospital | I could understand the reason and background behind the awareness of "participation" in rehabilitation and having students do a "life review" to improve the situation. |
| MD in a university hospital | I'm glad I participated because I was able to see outside of the perspective that medical professionals are trapped in. I realized that I have been trapped in the role of "doctor". |
| Pharmacist | It gave me a chance to think about what my purpose is as a pharmacist to be involved with patients. I can't stop thinking about what it means to be involved from a different perspective from that of a doctor, and what are the advantages of a different perspective. |
| Hospital pharmacist | It was very good to be able to share with everyone the doctor-patient relationship, the mind and body, and what the role of the doctor is. |
| Clinic | It was good to be able to deepen actual cases from various perspectives. I wanted to learn a little more about how to make use of it in the field and in medical treatment, as if deepening academic study is not something that can be directly returned to the field. |
| Not stated | It gave me an opportunity to think about the fundamental questions of medicine. |
| Other | I thought about the limits and possibilities of medicine. |

| | |
|---|---|
| Not stated | It was good to be able to look back from a new angle, not from medicine or psychology. |
| MD in a university hospital | I wondered if doctors are really objective. |
| MD in a hospital | I learned that an object is a possibility and a disease is "no longer possible. |
| MD in a hospital | It was good that I was able to talk about the things that bother me every day and see a bit of the perspective of anthropology, but I thought that if there was a take home message that I could remember when I was troubled even when I couldn't think about various difficult things, I could grab it and go home. |

201711 Fall

| Profile | Comments |
|---|---|
| MD in a hospital | I liked that there were many anthropologists and that interacting with doctors was a cross-cultural exchange in itself. |
| MD in a clinic | I realized that there is a gap between the language of the hospital, the language of the clinics and facilities, and the language of the patients and consumers. |
| NA | I was glad that there was an atmosphere that allowed me to talk about whatever I wanted. |
| MD in a clinic | It was interesting to mix the bug's eye view of case studies and the bird's eye view of culture in the discussion. |
| MD in a university | I can't find the words to use. I need to practice to enjoy the haze. |
| MD in a clinic and a hospital | I didn't know the word "medical anthropology" itself. More mumbo-jumbo leads to growth. |
| Other | I wanted more time. I was able to travel to explore people's perspectives |
| MD in a clinic | I learned how to accept difficult cases. |
| MD in a university | I was able to deepen the discussion based on specific common cases. |
| MD in a hospital | I wondered if I could use cultural anthropological ideas in my daily medical practice. |
| MD in a hospital | I could understand the difference between cultural anthropology and what I had learned on my own. |
| MD in a university | I had a chance to think about the state of scientific values. |
| Not stated | I wanted to learn about various topics in anthropology. |
| Not stated | I was able to hear perspectives and ideas that I had never heard before. |
| MD in a university | I liked the selection of cases. |
| MD in a clinic | It was good to be able to share the things that I usually dwell on but keep bottled up in the bottom of my heart, and to feel that a direction toward the future was being born, which is different from giving up. |
| MD in a private clinic | I would like you to verbalize the specifics of the involvement of cultural anthropology in medicine. |

| | |
|---|---|
| MD in a hospital | I wanted to learn more about medical science rather than medicine, but I was able to hear the opinions of those who study cultural anthropology. |
| MD in a clinic | When I heard that patients and their families have multiple hypotheses, I thought that I should listen to them more than ever while making hypotheses about their stories. |
| MD in a clinic and a hospital | I thought it would be connected to narrative based medicine as well as conflict resolution studies, etc. |

201801 Osaka Medical College

| Profile | Comments |
|---|---|
| MD in a clinic | It would have been nice to have the issues listed and presented on the white board. I think it would have been good to have the issues presented on a white board. I felt that it may be a problem of approach. |
| MD in a hospital | Difficulty in differentiating between autonomy and cognitive distortions. |
| MD in a clinic | It was good to learn about the value of considering medicine as a "foreign substance. |
| MD in a hospital | I felt that it is difficult to apply it in daily practice. |
| Other | I was able to learn how to bridge the differences in the perspectives of medical professionals and patients. |
